# Laser irradiation of ZnO:Al/Ag/ZnO:Al multilayers for electrical isolation in thin film photovoltaics

**DOI:** 10.1186/1556-276X-8-392

**Published:** 2013-09-23

**Authors:** Isodiana Crupi, Stefano Boscarino, Giacomo Torrisi, Giorgia Scapellato, Salvatore Mirabella, Giovanni Piccitto, Francesca Simone, Antonio Terrasi

**Affiliations:** 1 CNR-IMM MATIS, via S. Sofia 64, Catania 95123, Italy; 2 Dipartimento di Fisica e Astronomia, Università di Catania, via S. Sofia 64, Catania 95123, Italy; 3 CSFNSM, viale A. Doria 6, Catania 95125, Italy

**Keywords:** Transparent electrodes, Multilayers, Pulsed laser scribing, Thin film photovoltaics

## Abstract

Laser irradiation of ZnO:Al/Ag/ZnO:Al transparent contacts is investigated for segmentation purposes. The quality of the irradiated areas has been experimentally evaluated by separation resistance measurements, and the results are complemented with a thermal model used for numerical simulations of the laser process. The presence of the Ag interlayer plays two key effects on the laser scribing process by increasing the maximum temperature reached in the structure and accelerating the cool down process. These evidences can promote the use of ultra-thin ZnO:Al/Ag/ZnO:Al electrode in large-area products, such as for solar modules.

## Background

**Figure 1:**
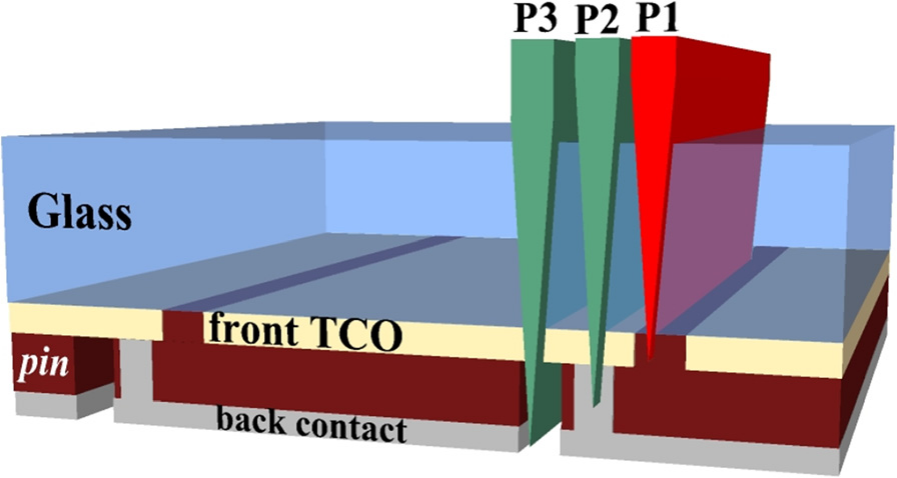
**Typical interconnect scheme of an α-Si:H module in superstrate configuration.** P1, P2 and P3 indicate the different patterning steps. P1 is performed using an infrared laser to remove the front TCO. P2 and P3 use a green laser to cut the Si solar absorber layer and the rear electrode, respectively.

Dielectric-metal-dielectric (DMD) multilayer structures are promising candidates for next-generation flexible transparent electrodes [1-4]. Compared to standard transparent conductive oxides (TCOs), DMD electrodes show enhanced conductivity, higher transmission of visible light, lower temperature process, reduced thickness and, consequently, significant cost reduction and improved mechanical flexibility [3,5-8]. For such advantages, DMD electrodes are frequently used in efficient optoelectronic devices including flat screen displays [9,10], organic light-emitting diodes (OLED) [11,12] and polymer solar cells (PSC) [13-15]. However, at present, DMD multilayer structures are still far from being implemented on thin film photovoltaic (TFPV) device technology. A crucial aspect is the film patterning process [16]. In the commercial production of hydrogenated amorphous silicon (α-Si:H), cadmium telluride (CdTe) and copper indium gallium di-selenide (CIGS) solar panels, the patterning method is accomplished by three laser scribing processes, also reported as P1, P2 and P3 [17]. These three steps allow the division of metre-sized solar panels into an array of smaller series interconnected cells [18,19], as illustrated in Figure 1. Specifically, the P1 scribe, with a laser wavelength of 1,064 nm, is used to segment the conductive coating on the glass into adjacent, electrically isolated stripes via ablation of the TCO layer. The P2 and P3 scribes, performed at 532 nm, cut the semiconductor layer and the rear electrode, respectively, via micro-explosions. So far, P1 laser scribing requires relatively high laser fluences and multipulse irradiation due to the optical transparency and mechanical hardness of the thick TCO (typically 0.7 to 1 μm), such as commercial SnO_2_:F (Asahi-U), In_2_O_3_:SnO_2_ (ITO) or ZnO:Al (AZO), currently used in thin film solar cells.

In this letter, we demonstrate how the energy density threshold for the scribing of the transparent contacts can be significantly reduced by replacing the standard thick AZO single layer with a 10 times thinner AZO/Ag/AZO multilayer structure with better electrical and optical properties. More specifically, for the lowest used pulse energy, we measure a separation resistance for the AZO/Ag/AZO structure 8 orders of magnitude higher compared to much thicker AZO, currently used in thin film solar cells. The experimental results and the numerical simulations provide clear evidences of the key role played by the silver interlayer to steep temperature increase at the DMD/glass interface, leading to a more efficient P1 scribing through a reduction of the fluence in a single laser pulse. These results could open great opportunities for the implementation of thin AZO/Ag/AZO electrodes on large-area modules liable to segmentation, such as for α-Si:H solar panels.

## Methods

AZO/Ag/AZO multilayers were sequentially deposited on conventional soda lime glass substrates by RF magnetron sputtering at room temperature in argon atmosphere with a working pressure of 1 Pa. A ceramic AZO target containing 2 wt.% Al_2_O_3_ and a pure Ag target were employed as source materials. The sputtering powers were 225 and 30 W for AZO and Ag, respectively. The deposition times were set in order to obtain 40 nm for both top and bottom AZO films and an optimum thickness of 10 nm for the Ag interlayer. This value was selected to fabricate a DMD structure that has high optical transparency in the visible range and good electrical conductivity [5]. The thicknesses of the films were verified by Rutherford backscattering spectrometry (RBS; 2.0-MeV He^+^ beam) measurements in normal detection mode. Laser treatments were performed in air by a single pulsed (12 ns) Nd:YAG laser operating with an infrared (*λ* = 1,064 nm), Gaussian-shaped (FWHM = 1 mm) beam. The laser power was varied to obtain fluences in the range from 1.15 to 4.6 J/cm^2^. The morphologies of the AZO/Ag/AZO multilayer after the laser irradiation process were investigated by field emission scanning electron microscopy (SEM) using a Zeiss Supra 25 microscope (Oberkochen, Germany). Electrical sheet resistance (*R*_sh_) of about 8 Ω/sq was measured on the as-deposited DMD electrode using a four-point terminal method by employing an HL5560 system (Bio-Rad, Hercules, CA, USA), while the change of the conductivity due to laser ablation process has been mapped by lateral current–voltage characteristics acquired with a Keithley 4200 semiconductor characterization system (Cleveland, OH, USA). Additionally, to simulate the laser process in our materials, a finite element method based on COMSOL Multiphysics software was employed.

## Results and discussion

**Figure 2:**
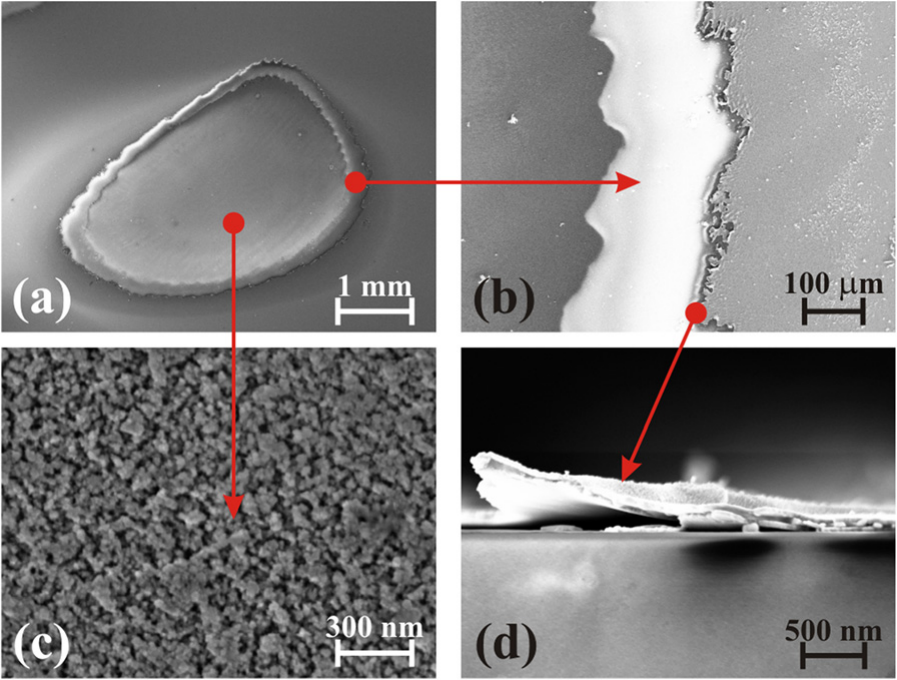
**SEM micrographs of the irradiated AZO/Ag/AZO electrode.** The laser irradiation is a single pulse, at a wavelength of 1,064 nm, duration of 12 ns and energy fluence of 1.7 J/cm^2^. The corresponding laser-irradiated spot size is 9.1 mm^2^. **(a)** Overview of the spot, **(b)** fracture of the multilayer structure at the periphery of the irradiated area, **(c)** central region and **(d)** AZO/Ag/AZO lift off from the substrate at the edge.

Figure 2 shows the SEM images of the AZO/Ag/AZO structure irradiated with a single laser pulse of 1.7 J/cm^2^. An irradiated region can be clearly observed in Figure 2a with no damage in the surroundings or cracking in the glass substrate. Figure 2b illustrates the well-defined cutting edges that leave the bare substrate uncovered with a flat and clean surface. It should be noted that both edges present modulated profiles such as the ones obtained if a laceration occurred. This quite large rip (approximately 200 μm wide) ensures an excellent isolation between the not irradiated DMD structure and the central area of the laser spot (see Figure 2c). Such an isolation is further guaranteed by the trilayer lift off from the substrate at the line border, as evident from the cross-sectional SEM image reported in Figure 2d.

**Figure 3:**
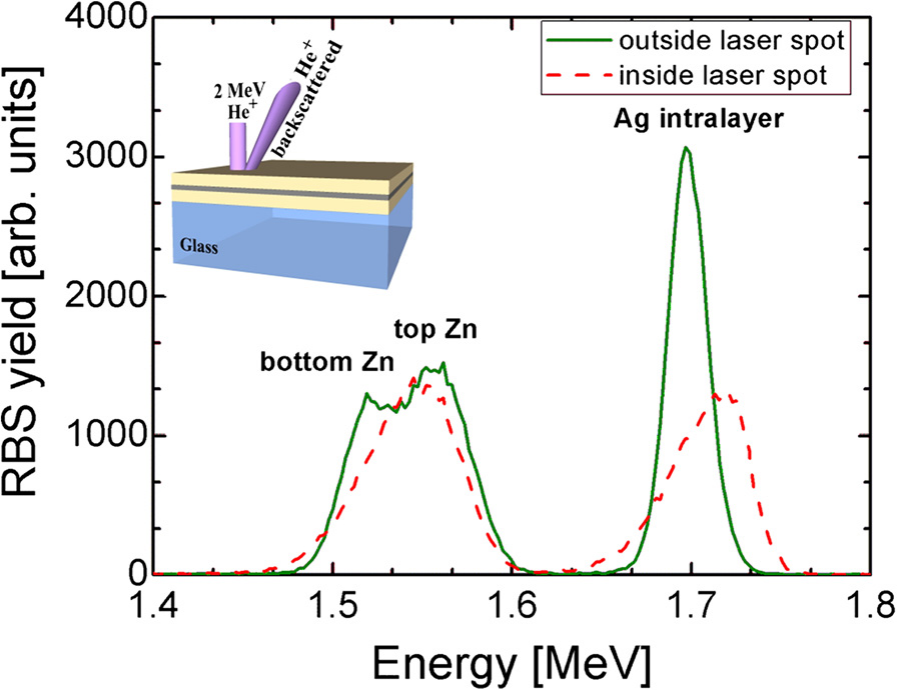
**Energy spectra of He**^**+ **^**backscattered by AZO/Ag/AZO samples outside and inside the irradiated area.** A scheme of the RBS experimental setup is reported in the inset.

The structural modification of the central area of the laser spot was confirmed by means of the RBS technique. Figure 3 compares the energy spectra of He^+^ backscattered by AZO/Ag/AZO samples outside and inside the irradiated region of Figure 2a. Three peaks are well distinguished in the as-deposited DMD. The one centred at 1.7 MeV is relative to He^+^ backscattered from Ag atoms, while the two peaks at 1.56 and 1.51 MeV are due to backscattering from the Zn atoms in the top and bottom AZO layers, respectively. Such a well-defined multilayer structure, present in the as-deposited DMD, disappears after laser irradiation, showing that both Ag and Zn atoms are now located at the surface (Ag signal shifted towards higher energy). The smaller area of Ag and Zn peaks after laser irradiation also indicates that a partial removal of these materials has occurred, while the broader shape of the signals is related to the loss of the sharp multilayer structure. This will have a noticeable effect on the electrical properties, as discussed in the following.

**Figure 4:**
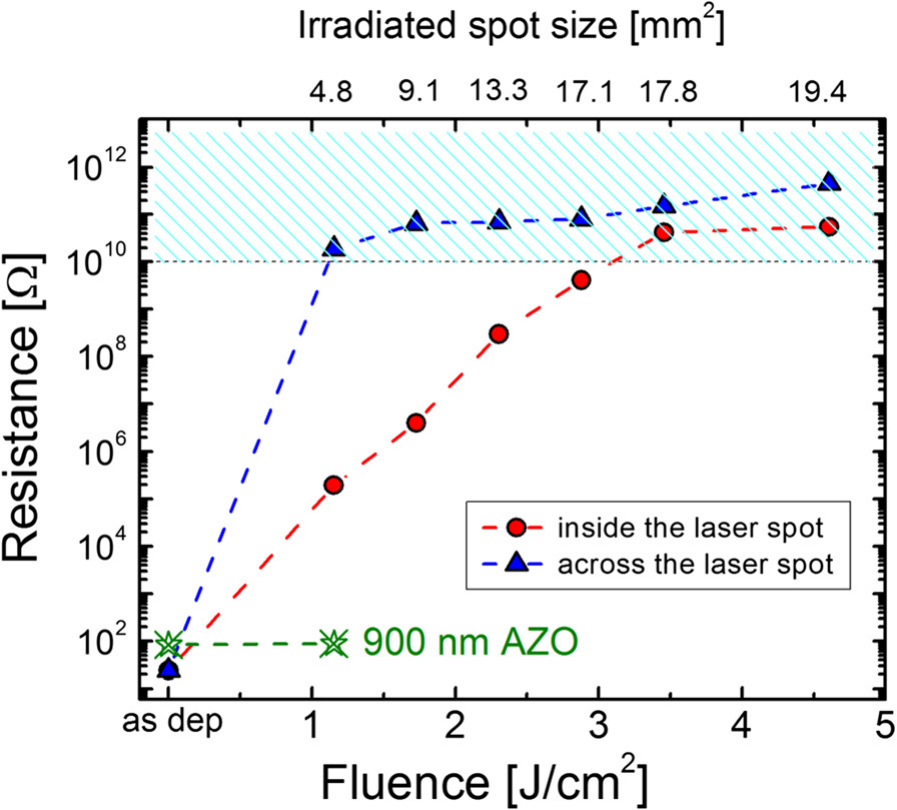
**Dependence of the separation resistance on laser fluences.** The irradiated spot size enlargement, evaluated through SEM imaging, is reported on the top *x*-axis. The cyan dashed area corresponds to the situation of excellent separation resistances (**≥**10 MΩ).

Figure 4 shows the separation resistance measured between two points, at a distance of 1.2 mm from each other, inside and across the laser spot, on our thin AZO/Ag/AZO sample irradiated with various laser fluences. With the increase of the pulse energy, the resistance measured inside the laser spot continuously increases up to saturation above 10 MΩ reached for fluence values higher than 3 J/cm^2^ ensuring a complete electrical insulation. This is due to the more efficient ablation and damage of the film with the laser power, as also indicated by the spot area reported in the top *x*-axis scale. The increase of the laser fluence implies a steeper temperature gradient across the multilayers resulting in a damage of the DMD structure, thus, in an electrical insulation, more and more pronounced. Most interestingly, the measured resistance values across the edge of the laser spot show an excellent insulation even at the lowest used beam fluence with an increase, with respect to the as-deposited multilayers, of more than 8 orders of magnitude. Such high separation resistance is maintained also for higher laser fluences and can be attributed to the occurrence of the DMD laceration, as showed in Figure 2b. Similar separation resistance was not observed in the case of a reference thick AZO layer, irradiated under the same condition and included in Figure 4 for comparison. To understand how the separation resistance can be related to the laceration, a further description of the DMD irradiation process is needed.

The DMD removal process with nanosecond pulse irradiation occurs in three consecutive steps: absorption of the laser energy at the transparent electrode/glass interface, steep temperature increase of the irradiated area, and fracture and damage of the continuous conductive multilayers. To accurately describe this process, a thermal model was applied [20]. The time-dependent temperature distribution in the irradiated samples is calculated according to the heat conduction equation:

(1) $$\rho C_{p}\left(\frac{\mathrm{\delta T}}{\mathrm{\delta t}}+v_{\mathrm{rec}}\nabla T\right)=\nabla \cdot \left(\kappa \nabla T\right)+\Sigma ,$$

where *ρ*, *C*_p_ and *κ* are the mass density, the thermal capacity and the thermal conductivity of the material, respectively. The recession velocity, *v*_rec_, is neglected in view of relatively low laser fluences which are insufficient for heating of the considered materials above the melting threshold and, thus, to initiate thermal vaporization [17]. The laser source term is given by

(2) $$\Sigma =Q\left(x,y\right)\left(1-R\right)\alpha e^{\left(-\mathrm{\alpha z}\right)}f\left(t\right),$$

where *α* and *R* are the absorption and reflection coefficients of the material, respectively. *Q*(*x*,*y*) is the incident laser pulse intensity with a Gaussian spacial profile, and *f*(*t*) is the square-shaped pulse in the time domain:

(3) $$f\left(t\right)=\left\{\begin{array}{l} 1\begin{array}{cc} & 0<t<12\;\mathrm{ns} \end{array}\\ 0\begin{array}{cc} & \mathrm{otherwise}. \end{array} \end{array}\right)$$

Equation 1 is calculated for each layer of the structure using the material properties summarized in Table 1.

**Table 1:** **Material properties used in Equation 1**[21-23]

**Parameters**	**Material**	**Value**
Specific heat, *C*_p_ (J kg^−1^ K^−1^)	Glass	703
	Ag	240
	AZO	494
Density, *ρ* (g cm^−3^)	Glass	2.2
	Ag	10.49
	AZO	5.7
Thermal conductivity, *κ* (W m^−1^ K^−1^)	Glass	0.80
	Ag	429
	AZO	20
Absorption coefficient, *α* (cm^−1^) (at 1,064 nm)	Glass	0.5
	Ag	1.03 × 10^5^
	AZO	4 × 10^3^
Reflection coefficient, *R* (at 1,064 nm)	Glass	0.04
	Ag	0.64
	AZO	0.01

**Figure 5:**
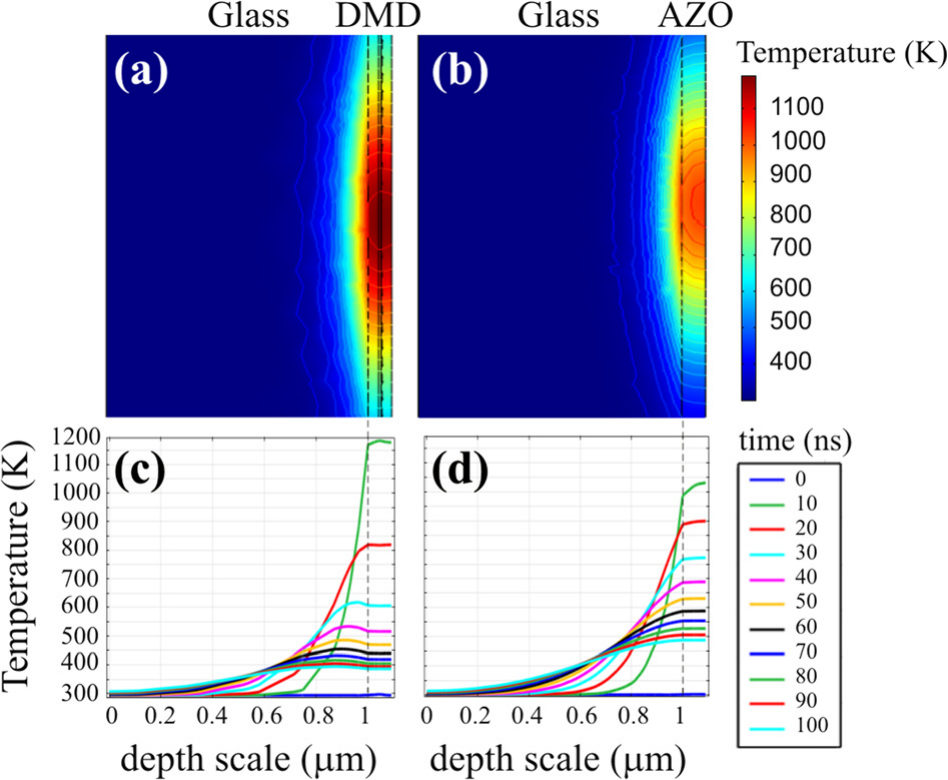
**Simulations of the thermal process.** Temperature distribution on 40-nm AZO/10-nm Ag/40-nm AZO on glass **(a**, **c)** and on 90-nm AZO on glass **(b**, **d)**. The laser irradiation is a single pulse, at a wavelength of 1,064 nm, duration of 12 ns and energy fluence of 1.15 J/cm^2^.

Figure 5 shows the simulations of the thermal process (in *XZ*-plane) on two samples irradiated with a single pulse, at a wavelength of 1,064 nm, duration of 12 ns and the lowest used fluence of 1.15 J/cm^2^. The samples (both 90 nm thick on glass substrates) differ only for the presence of a 10-nm Ag mid-layer and are initially at room temperature. Interestingly, immediately after the laser pulse, the maximum temperature reached in the multilayer structure is 150 K higher than that in the single AZO film, probably due to the higher absorption coefficient of the noble metal material at this wavelength. This is also indicated by the temperature distribution centred at the Ag depth in Figure 5a with respect to Figure 5b where the highest value is located at the surface of the AZO film. The same can be claimed by observing the spatio-temporal curves, reported in Figure 5c,d. Here, the green lines indicate the temperature values after 10 ns from the beginning of the laser pulse, and it is clear as the temperature is higher for the DMD sample and how the maximum value coincides with the Ag location, whereas this is not the case for the single AZO film. Also, the evolution of temperatures with time is quite different for the two samples, with a faster cooling after the laser process for the multilayer sample. Such a behaviour can be related to the higher thermal conductivity of Ag with respect to AZO. In addition, the simulations performed on a 10 times thicker AZO film (not reported here) show that the maximum temperature reached after the laser pulse is similar to the ultra-thin DMD structure, but the cool down process is even slower. These observations indicate that a 10-nm-thin Ag mid-layer greatly affects the heat flow during and after the laser irradiation, with noticeable effects on film removal thresholds. In fact, we experimentally observed that for DMD thin film, a much lower laser energy fluence is required to induce the film cracking.

## Conclusions

A single nanosecond laser pulse has been used to investigate the scribing process of an ultra-thin DMD electrode (AZO/Ag/AZO structure). Given a reduced pulse energy of 1.15 J/cm^2^, the separation resistance of AZO/Ag/AZO is enhanced by 8 orders of magnitude compared to thicker AZO, currently used in thin film solar cells. The thermal behaviour, simulated using a finite element approach, shows that the silver interlayer plays two key effects on the scribing process by increasing the maximum temperature reached in the structure and fastening the cool down process. It is worth noting that although only a partial ablation of the DMD occurs at low laser fluences, the presence of the rip at the edge of the spot ensures an excellent electrical isolation, while such a morphology in standard TCO upon laser processing has never been reported to our knowledge. The presence of Ag has two main effects on the laser process: (1) higher temperature gradients and (2) different expansion and contraction of each layer during and after the irradiation, respectively. The latter point is a consequence not only of the first one (high thermal gradient between glass and film) but also of the difference in the thermal expansion coefficients of the materials: 18.9 × 10^−6^, 4.75 × 10^−6^ and 8.9 × 10^−6^ K^−1^ for Ag, AZO and soda lime, respectively. The substrate and coatings will expand differently upon the temperature change during the laser irradiation. As a result, thermally induced stresses are expected to arise. Because of the lower thermal expansion coefficient, AZO layers will suffer a reduced expansion with respect to the inner Ag film, and a compressive stress is then exerted by the inner layer on the outer layers which, after the thermal quenching, gives birth to the observed laceration. Our results, in combination with its excellent electro-optical properties, make the AZO/Ag/AZO electrode a suitable candidate for use in large-area modules, liable to segmentation, such as for α-Si:H solar panels.

## Competing interests

The authors declare that they have no competing interests.

## Authors’ contributions

IC contributed to the sample processing, characterization, data analysis and interpretation and drafted the manuscript. SB synthesized the samples and contributed to the sample characterization and data analysis. GT and GP provided the simulation data. GS carried out the laser treatments. SM performed the RBS characterization and contributed to the data interpretation. FS contributed to the optical analysis. AT conceived the study and contributed to the data interpretation. All authors read and approved the final manuscript.
